# Approximate Nearest Neighbor Search by Residual Vector Quantization

**DOI:** 10.3390/s101211259

**Published:** 2010-12-08

**Authors:** Yongjian Chen, Tao Guan, Cheng Wang

**Affiliations:** 1 Digital Engineering & Simulation Research Center, Huazhong University of Science and Technology, Wuhan 430074, China; E-Mails: wangch@hhu.edu.cn (Y.C.); chyojn@gmail.com (C.W.); 2 School of Computer Science & Technology, Huazhong University of Science and Technology, No.1037 Luoyu Road, Wuhan 430074, China

**Keywords:** approximate nearest neighbor search, high-dimensional indexing, residual vector quantization

## Abstract

A recently proposed product quantization method is efficient for large scale approximate nearest neighbor search, however, its performance on unstructured vectors is limited. This paper introduces residual vector quantization based approaches that are appropriate for unstructured vectors. Database vectors are quantized by residual vector quantizer. The reproductions are represented by short codes composed of their quantization indices. Euclidean distance between query vector and database vector is approximated by asymmetric distance, *i.e.*, the distance between the query vector and the reproduction of the database vector. An efficient exhaustive search approach is proposed by fast computing the asymmetric distance. A straight forward non-exhaustive search approach is proposed for large scale search. Our approaches are compared to two state-of-the-art methods, spectral hashing and product quantization, on both structured and unstructured datasets. Results show that our approaches obtain the best results in terms of the trade-off between search quality and memory usage.

## 1. Introduction

Approximate nearest neighbor search (ANN) is proposed to tackle the curse of the dimensionality problem [1,2] in exact nearest neighbor (NN) searching. The key idea is to find the nearest neighbor with high probability. ANN is a fundamental primitive in computer vision applications such as keypoint matching, object retrieval, image classification and scene recognition [3]. In many computer vision applications, the data-points are high-dimensional vectors that are embedded in Euclidean space, and the memory usage for storing and searching high-dimensional vectors is a key criterion for problems involving large amount of data.

The state-of-the-art approaches such as tree-based methods (e.g., KD-tree [4], hierarchical k-means (HKM) [5], FLANN [6]) and hash-based methods (e.g., Exact Euclidean Locality-Sensitive Hashing (E2LSH) [7,8]) involve indexing structures to improve the performance. The memory usage of indexing structure may even be higher than the original data when processing large scale data. Moreover, FLANN and E2LSH need a final re-ranking based on exact Euclidean distance, which means the original vector should be stored in main memory, this requirement seriously limits the databases’ scale. Binary index methods such as [9–11] simplify the indexing structure by using binary code to index the space partitions. However, these methods also need the original vector for final re-ranking.

Recently proposed hamming embedding methods compress the vectors into short codes and approximate the Euclidiean distance between two vectors by the hamming distance between their codes. These methods include hamming embedding [12], miniBOF [13], small hashing code [14], small binary code [15] and spectral hashing [16]. These methods make it possible to store large scale data in main memory. One weakness of these methods is the discrimination limitation of hamming distance as the total number of possible hamming distance is limited by code length. [17] introduced product quantization to compress the vector into several bytes and proposed a more accurate distance approximation. However, its search quality is limited on unstructured vector data.

Objectives of the paper are comparable to those of [16,17]: (1) storing millions of high-dimensional vectors in memory and (2) quickly finding similar vectors to a target vector. In contrast with product quantization, we focus on the performance for unstructured vector data. We introduce residual vector quantization, which is appropriate for unstructured data, for the vector encoding. An efficient exhaustive search method is proposed based on fast distance computing. A non-exhaustive search method is proposed to improve the efficiency for large scale search. Our approaches are compared to two state-of-the-art methods, spectral hashing and product quantization, on both structured and unstructured datasets. Results show that our approaches obtain the best results in terms of accuracy and speed.

Our paper is organized as follows: Section 2 presents the residual vector quantization and Section 3 introduces our exhaustive and non-exhaustive search methods that are based on the residual vector quantization. Section 4 evaluates the search performance and compares our approaches with two state-of-the-art methods. Section 5 discusses the results and Section 6 is the conclusion.

## 2. Residual Vector Quantization

A *K*-point vector quantizer*Q* maps a vector *x*∈*R^D^* into its nearest centroidin codebook *C* = {*c_i_*, *i* = 1..*K*} ⊂ *R^D^*:

(1) $$\tilde{x}=Q(x)=\underset{c_{i}\in C}{\text{arg}\;\text{min}}\;d(x,\;c_{i})$$

where *d*(*x*, *c_i_*) is the exact Euclidean distance between *x* and *c_i_*. This destructive process can be interpreted as approximating the *x* by one of centroids in *R^D^* space [18], and the residual vector is:

(2) $$ɛ=x-\tilde{x}=x-Q(x)$$

The performance of quantizer *Q* is measured by mean squared error (MSE):

(3) $$\text{MSE}(Q)=\text{EX}\left[d{\left(x,\;Q(x)\right)}^{2}\right]$$

**Figure 1.**
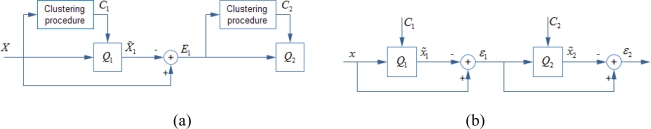
Block diagrams of two-stages residual vector quantization. **(a)** Learning codebooks; **(b)** Quantizing a vector.

Residual vector quantization [19,20] is a common technique to reduce the quantization error with several low complexity quantizers. Residual vector quantization approximate the quantization error by another quantizer instead of discard it. Several stage-quantizers, each has its corresponding stage-codebook, are connected sequentially. Each stage-quantizer approximates preceding stage’s residual vector by one of centroids in the stage-codebook and generates a new residual vector for succeeding quantization stage. Block diagrams of a two stages residual vector quantization are shown in Figure 1. In the learning phase (Figure 1(a)), a training vector set *X* is provided and the first stage-codebook *C_1_* is generated by k-means clustering method. The entire training set is then quantized by the first stage-quantizer *Q_1_* which is defined by *C_1_*. The difference between *X* and its first stage quantization outputs, which is the first residual vector set E_1_, is used for learning the second stage-codebook *C_2_*. In quantizing phase (Figure 1(b)), the input vector *x* is quantized by first stage-quantizer *Q_1_*, which is defined by first stage-codebook *C_1_*. The difference between *x* and its first stage quantization output, which is the first residual vector *ɛ_1_*, is quantized by second stage-quantizer *Q_2_*. The second residual vector *ɛ_2_* is discarded. The first two quantization outputs are used to approximate the input vector:

(4) $$x={\tilde{x}}_{1}+{ɛ}_{1}={\tilde{x}}_{1}+{\tilde{x}}_{2}+{ɛ}_{2}\approx {\tilde{x}}_{1}+{\tilde{x}}_{2}=\tilde{x}$$

For *L* stages residual vector quantization, a vector *x* is approximated by the sum of its *L* stages’ quantization outputs while the last stage’s quantization error is discarded:

(5) $$x=\sum_{i=1}^{L}{\tilde{x}}_{i}+{ɛ}_{L}\approx \sum_{i=1}^{L}{\tilde{x}}_{i}=\tilde{x}$$

For transformation or storage, indices of quantization outputs are used. For *L* stage residual vector quantization, which is constructed by *K*-point vector quantizers, the bit rate is *L* log_2_ *K* per vector.

The quantization performance of *i*th stage-quantizer is:

(6) $$\text{MSE}(Q_{i})=\frac{1}{N}\sum_{ɛ\in {\text{E}}_{i}}{ɛ}^{T}ɛ=\frac{1}{N}\sum_{j=1}^{K}\sum_{x\in V_{j}}{\left\|x-c_{i,j}\right\|}^{2}$$

where E*_i_* is the new residual vector set generated by *Q_i_*, *V_j_* is the *j*th cluster and *c_i_*_,_*_j_* is *V_j_*’s centroid.

Considering the optimization problem of finding a vector *y* to minimize the objection function:

(7) $$J=\sum_{x\in V_{j}}{\left\|x-y\right\|}^{2}$$

By differentiating the objection function *J* with respect to *y* and setting derivative equal to zero, it is easy to obtain the minimizing *y*:

(8) $$y=\frac{1}{N_{j}}\sum_{x\in V_{j}}x$$

where *N_j_* is the number of vectors in *j*th cluster. This means the centroid of cluster minimizes the objection function:

(9) $${\sum_{x\in V_{j}}{\left\|x-c_{i,j}\right\|}^{2}={\text{min}}_{y}\sum_{x\in V_{j}}{\left\|x-y\right\|}^{2}\leq \sum_{x\in V_{j}}{\left\|x-y\right\|}^{2}|}_{y=0}=\sum_{x\in V_{j}}{\left\|x\right\|}^{2}$$

With the observations that $\sum_{ɛ\in {\text{E}}_{i}}{ɛ}^{T}ɛ=\sum_{j=1}^{K}\sum_{x\in V_{j}}{\left\|x-c_{i,j}\right\|}^{2}$ and $\sum_{x\in {\text{E}}_{i-1}}x^{T}x=\sum_{j=1}^{K}\sum_{x\in V_{j}}{\left\|x\right\|}^{2}$, we obtain the inequality:

(10) $$\text{MSE}(Q_{i})\leq \text{MSE}(Q_{i-1})$$

which means the k-means clustering method guarantee the MSE of stage-quantizers are decreasing monotonically.

## 3. Using Residual Vector Quantization for ANN

### 3.1. Exhaustive Search by Fast Distance Computation

In [17] the exact Euclidean distance between two vectors is approximated by asymmetric distance, *i.e.*, the distance between a vector and a reproduction of another vector:

(11) $$d(x,\;y)\approx \tilde{d}(x,\;y)=d(x,\;Q(y))$$

Asymmetric distance reduces the quantization noise and improves the search quality [17]. We have proposed fast asymmetric distance computation based on residual vector quantization. Suppose a database vector *y* is quantized by *L* × *K* residual vector quantizer, its indices of quantization output are {*u_j_*, 1 ≤ *u_j_* ≤ *K*, *j* =1..*L*}, and the reproduction of *y* is constructed by the sum of corresponding centroids:

(12) $$\tilde{y}=\sum_{i=1}^{L}{\tilde{y}}_{i}=\sum_{i=1}^{L}c_{i,u_{i}},\;c_{i,u_{i}}\in C_{i},\;1\leq u_{i}\leq K$$

where *c_i,u_i__* is the *u_i_*th centroid of codebook *C_i_*. The squared asymmetric distance between *y* and the target vector *x* is the exact squared distance between *x* and *ỹ*:

(13) $$\begin{array}{l} \tilde{d}{\left(x,\;y\right)}^{2}=d{\left(x,\;\tilde{y}\right)}^{2}={\left\|x-\tilde{y}\right\|}^{2}={\left\|x\right\|}^{2}+{\left\|\tilde{y}\right\|}^{2}-2\langle x,\;\tilde{y}\rangle \\ ={\left\|x\right\|}^{2}+{\left\|\tilde{y}\right\|}^{2}-2\langle x,\;\sum_{i=1}^{L}c_{i,u_{i}}\rangle ={\left\|x\right\|}^{2}+{\left\|\tilde{y}\right\|}^{2}-2\sum_{i=1}^{L}\langle x,\;c_{i,u_{i}}\rangle \end{array}$$

where 〈*x*, *y*〉 is dot product. ||*ỹ*|| is pre-computed off-line when the database vector is quantized. The dot products of codebooks’ centroids and target vector *x* are computed and stored in a look-up table when *x* is submitted:

(14) $$T=\left\{t_{i,j}\right\},\;t_{i,j}=\langle x,\;c_{i,j}\rangle ,\;c_{i,j}\in C_{i},1\leq i\leq L,\;1\leq j\leq K$$

The squared asymmetric distance can then be efficiently estimated by several table lookups:

(15) $$\tilde{d}{\left(x,\;y\right)}^{2}={\left\|x\right\|}^{2}+{\left\|\tilde{y}\right\|}^{2}-2\sum_{i=1}^{L}t_{i,u_{i}}$$

If we only consider the order of distance, term ||*x*|| is a constant for all database vector and can be ignored in asymmetric distance computation. *R* nearest neighbors are selected based on the estimated squared asymmetric distances.

### 3.2. Non-Exhaustive Search by Rough Approximation

Exhaustive search has to scan quantization codes of all database vectors. In problems such as bag-of-features-based large scale image retrieval, billions of images are represented by hundreds of local feature vectors per image, and it is prohibitive to scan the feature vector database, even with fast asymmetric distance computation.

In [17] the authors proposed a non-exhaustive search method for large scale datasets. A coarse quantizer is involved to filter out farther database vectors, and then a product quantizer is used for fine search. In contrast with using an external coarse quantizer, we propose a straight forward non-exhaustive search approach based on the approximating sequence of database vector *y* that is generated by residual vector quantization:

(16) $$\left\{{\tilde{y}}^{\left(l\right)}\right\},\;{\tilde{y}}^{\left(l\right)}=\sum_{i=1}^{l}{\tilde{y}}_{i},\;1\leq l\leq L$$

Our exhaustive search approach uses only the most accurate item *ỹ*^(^*^L^*^)^ to approximate the *y*. In non-exhaustive search, the first *L*_1_ quantization outputs generate a rough approximation:

(17) $${\tilde{y}}^{\left(L_{1}\right)}=\sum_{i=1}^{L_{1}}{\tilde{y}}_{i},\;L_{1}<L$$

The rough asymmetric distances between database vectors and the target vector are then evaluated by table lookups for coarse search:

(18) $$d{\left(x,\;{\tilde{y}}^{\left(L_{1}\right)}\right)}^{2}={\left\|x\right\|}^{2}+{\left\|{\tilde{y}}^{\left(L_{1}\right)}\right\|}^{2}-2\sum_{i=1}^{L_{1}}t_{i,u_{i}}$$

The database vectors which have large rough distances are pruned and the remaining database vectors are used to evaluate more accurate distances to the target vector by their most accurate approximations as in Equation (13).

The total number of possible rough approximations is *K*^*L*_1_^, thus an inverted file system is used to improve the search performance. Each inverted list corresponds to a possible rough approximation. When encoding database vectors by *L* × *K* residual vector quantization, each vector’s first *L*_1_ indices are used to determine which inverted list it should be inserted in, then the *L*_1_ indices are discarded and only the last *L*_2_ = *L − L*_1_ indices and its vector id are stored in the inverted list. A query vector first evaluated its distances to the *K*^*L*_1_^ possible rough approximations by Equation (18). The *W* nearest rough approximations are selected and corresponding *W* inverted lists are scanned to evaluate more accurate distance to query vector:

(19) $$\begin{array}{l} d{\left(x,\;{\tilde{y}}^{\left(L\right)}\right)}^{2}={\left\|x\right\|}^{2}+{\left\|{\tilde{y}}^{\left(L\right)}\right\|}^{2}-2\langle x,\;{\tilde{y}}^{\left(L\right)}\rangle ={\left\|x\right\|}^{2}+{\left\|{\tilde{y}}^{\left(L\right)}\right\|}^{2}-2\sum_{i=1}^{L}\langle x,\;c_{i}^{\left(u_{i}\right)}\rangle \\ =d{\left(x,\;{\tilde{y}}^{\left(L_{1}\right)}\right)}^{2}+{\left\|{\tilde{y}}^{\left(L\right)}\right\|}^{2}-{\left\|{\tilde{y}}^{\left(L_{1}\right)}\right\|}^{2}-2\sum_{i=L_{1}-1}^{L}\langle x,\;c_{i}^{\left(u_{i}\right)}\rangle \end{array}$$

Equation (19) shows the squared asymmetric distances which are computed in fine search can be updated by squared rough distance in the coarse search and only *L*_2_ table lookups per vector are involved. The term ||***ỹ***^(***L***)^||^2^ – ||***ỹ***^(***L*_1_**)^||^2^ is pre-calculated and stored in offline quantization stage. By fast table lookups and distance update scheme, both coarse and fine search are efficient. *R* nearest neighbors are selected based on the squared asymmetric distances that are estimated in fine search.

## 4. Experiments and Results

### 4.1. Dataset

Three public available datasets were used to evaluate the performances of ANN methods: the structured SIFT descriptor dataset [21], semi-structured GIST descriptor dataset [21] and unstructured VLAD descriptor dataset [22]. SIFT descriptor codes small image patch while GIST descriptor and VLAD descriptor code entire image. SIFT descriptor is a histogram of oriented gradients that extracted from gray image patch. GIST descriptor is similar to SIFT applied to the entire image. It applies an oriented Gabor filter over different scales and averages the filter energy in each bin. The VLAD descriptor is constructed by first aggregating images’ SIFT descriptors’ quantization residual vectors locally and then reducing their dimensions by PCA.

The SIFT dataset and GIST dataset have three subsets: learning set, database set, and query set. The learning set is used for learning the model and evaluating quantization performance, the database and query sets are used for evaluating ANN search performance. For the SIFT dataset, the learning set is extracted from Flicker images [12] and the database and query descriptors are from INRIA Holidays images [23]. For GIST, the learning set consists of a subset of the tiny image set of [24]. The database set is the Holidays image set combined with Flicker1M used in [12]. The query vectors are from the Holidays image queries [23]. VLAD dataset is generated by public package and public local image descriptors [22] which are extracted from Holiday image dataset [23]. The dataset has 1,491 128-dimensional vectors and was divided into 500 groups. The first descriptor of each group is the query image and the correct retrieval results are the other images of the group. Total vectors in dataset are used as training set and database set. All these descriptors are high-dimensional float vectors. Scales of these datasets are summarized in Table 1.

### 4.2. Quantization Performance

This section investigates the quantization performance of our approach by evaluating the influence of parameters over quantization error. *K* is the number of centroids of stage-quantizer, *L* is the total number of stage-quantizers. The code length, *i.e.*, *L* log_2_ *K*, is regarded as a metric of storage.

**Figure 2.**
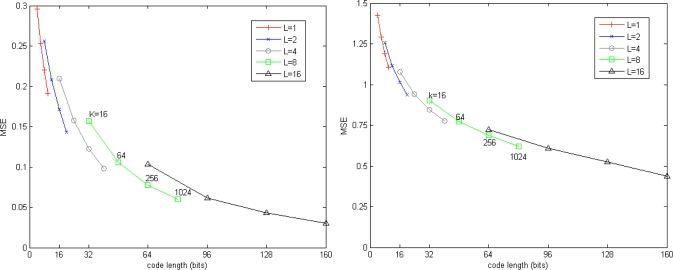
Quantization error associated with *K* and *L*. (**left**) SIFT dataset; (**right**) GIST dataset.

Figure 2 shows the trade-offs between quantization accuracy and memory. It is clear that the quantization error is reduced by increase either *K* or *L*. For a fixed number of bits, the residual vector quantizer which has fewer stage-codebooks and more centroids in each stage-codebook is more accurate than the residual vector quantizer which has more stage-codebooks and fewer centroids in each stage-codebook.

### 4.3. Parameters’ Influences on Search Accuracy

The performances of our approaches are measured by two metrics: recall@R and ratio of distance errors (RDE). Recall@R is defined in [17] as the proportion of query vectors for which the nearest neighbor is randked in the first R positions. Values of recall@R close to 1 indicate high quality of search results. RDE [11] is defined as:

(20) $$\text{RDE}=1-\frac{\sum_{i=1}^{k}d({\text{NN}}_{i},\;x)}{\sum_{i=1}^{k}d({\text{ANN}}_{i},\;x)}$$

where *NN_i_* is the *i*th exact nearest neighbor of query *x* and *ANN_i_* is *x*’s *i*th approximate nearest neighbor. Values of RDE close to 0 indicate high quality of results. Mean and standard variance of RDE is used to measure the average search quality.

**Figure 3.**
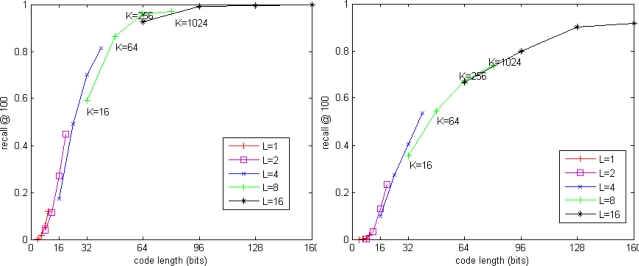
Exhaustive search accuracy. (**left**) SIFT dataset. (**right**) GIST dataset.

**Figure 4.**
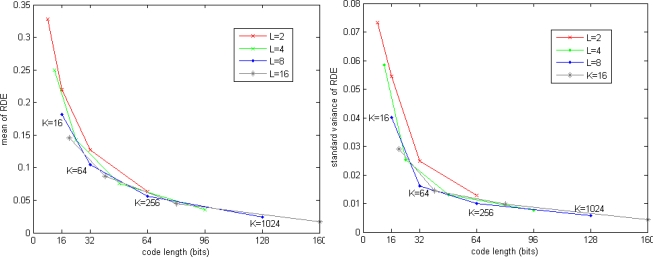
RDE for SIFT dataset, exhaustive search method. (**left**) mean of RDE. (**right**) standard variance of RDE.

Figure 3 and 4 show the performance of our exhaustive search method. Figure 3 shows the trade-off between recall@R and code length for SIFT and GIST datasets. When the code length is fixed, the residual vector quantizer which has fewer codebooks and more centroids in each codebook is more accurate than the residual vector quantizer which has more codebooks and fewer centroids in each codebook. It seems a good choice to use 8 × 256 residual vector quantization for SIFT descriptor and 16 × 256 residual vector quantization for GIST descriptor.

Figure 4 shows the RDE for SIFT dataset. The mean of RDE is tending to 0 when increasing code length. The standard variance of RDE is also significant reduced when increasing code length, which means the query results are more stable when more bits are used to encode the vectors.

**Figure 5.**
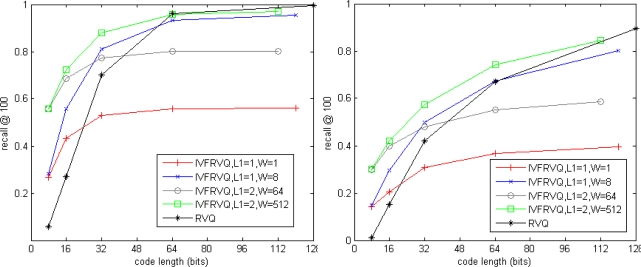
Search accuracy of non-exhaustive search. (**left**) SIFT dataset. (**right**) GIST dataset.

Figure 5 shows impact of the parameters for our non-exhaustive search method. *K =* 256, *L*_1_ ∈ {1,2} and *L*_2_ ∈ {1,2,4,8,16} are the numbers of stage-quantizers used for coarse search and fine search, *W* is the number of candidate inverted lists for fine search. The total number of inverted lists is *K*^*L*_1_^. The code length *L*_2_ log_2_ *K* is regarded as a metric of storage. Results of our exhaustive search method are also plotted in dash line for comparison. For simplicity, our exhaustive search and non-exhaustive search methods are respectively denoted as RVQ and IVFRVQ. We observed that the performance of IVFRVQ strongly depends on *W* which determines the fraction of inverted lists that are scanned. When a small fraction of inverted lists are scanned, increasing the code length is useless for improving the performance. When sufficient inverted lists are scanned, performance of IVFRVQ is comparable to even better than RVQ.

Tables 2 and 3 show comparisons of search efficiency. Both RVQ and IVFRVQ encode the vector into 64-bit code. It is clear that the pruning strategy significantly reduces the search time. It is noticed that it has to increase the *W* for search accuracy when *L*_1_ = 2, but the frequent inverted lists access reduces the search performance.

### 4.4. Compared with the State of the Art

In this section we compare our approach with two state-of-the-art methods: spectral hashing (SH) and product quantization. The performance of product quantization is sensitive to the grouping order of vector components. The natural product quantization groups the consecutive components while the structured product quantization groups related components together based on the prior knowledge of vector’s structure. Experimental results in [17] show that the natural product quantization is appropriate for SIFT descriptor while the structured product quantization is appropriate for GIST descriptor. For simplicity, the natural product quantization method is denoted as PQ while the structured product quantization method is denoted as PQ*, their non-exhaustive version are denoted as IVFPQ and IVFPQ* respectively. Vectors are compressed into 64-bit binary codes. Eight 256-point quantizers are used for PQ and a 1024-point quantizer is used as the coarse quantizer for IVFPQ. We use *L* = 8, *K* = 256 for RVQ and *L*_1_ = 1, *L*_2_ = 8, *K* = 256 for IVFRVQ.

**Figure 6.**
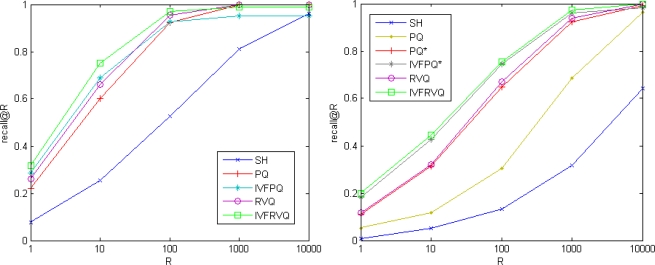
Comparison of search accuracies obtained by spectral hashing, product quantization methods and our approaches. (**left**) SIFT dataset, 64-bit codes. (**right**) GIST dataset, 64-bit codes.

Figure 6 compares the search qualities on SIFT and GIST datasets. On the benchmark SIFT, our approaches significantly outperform spectral hashing and are slightly better than product quantization methods. On the benchmark GIST, our approaches significantly outperform spectral hashing and natural product quantization methods and are comparable to structured product quantization methods.

The VLAD dataset is used for evaluating the accuracy of ANN methods on unstructured vectors. The performance is measured by mean average precision (mAP) [22] which is defined as the area of recall-precision curve, a larger value of mAP indicate a better retrieval performance. Table 4 shows the accuracies obtained by different methods (spectral hashing, product quantization and our approach) and different code length configurations (32 bits, 64 bits, 128 bits). Both product quantizer and our residual vector quantizer are constructed by 256-point vector quantizer. The code length of spectral hashing is directly assigned while those of product quantization and our approach are controlled by the number of quantizers. We use a 1024-point quantizer as the coarse quantizer for IVFPQ. We only test the 32-bit and 64-bit configurations for our approaches because the stage-quantization errors are too small to be handled by our single precision implementation when 16 stage-quantizers are used. It is clear that our approach is significant outperform spectral hashing and product quantization. Equivalently, our method obtains a comparable search quality with only half the code length of product quantization.

### 4.5. Speed Comparison

Table 5 compares the search time of different methods on the SIFT dataset. Spectral hashing and product quantization use the public available Matlab packages. Our approaches are implemented in Matlab. Both the hamming distance computation for spectral hashing and the asymmetric distance computation for product quantization and our approaches are optimized by C. All methods compress SIFT descriptors 64-bit binary code. The time is measured on a 2.2 GHz CPU laptop with 3 GB of RAM. The approaches RVQ, PQ and SH have similar rum times because they all scan the whole database and compute the distances by table lookups. Non-exhaustive search methods significant improve the performance. IVFRVQ is more efficient than IVFPQ for equal search accuracy because IVFPQ calculates *W* look-up tables for individual candidate inverted list while IVFRVQ only calculates one look-up table.

## 5. Discussion

### 5.1. Advantages of Residual Vector Quantization

The advantage of residual vector quantization is quantizing the whole vector in original space. Product quantization is based on the assumption that the subspaces are statistically mutual independent such that the original space can be represented by the production of these subspaces. But vectors in real data do not all meet that assumption. Moreover, the vector’s structure determines the quantization parameters and makes product quantization inflexible. In contrast, residual vector quantization processes the whole vector in original space, and the parameters are not limited by the structure of vector.

### 5.2. Link between Residual Vector Quantization and Hierarchical k-means

Residual vector quantization can be regarded as a simplified hierarchical k-means (HKM). When generating a new quantization level, HKM performs k-means clustering in each previous level’s cluster and generate a new partition for each previous level’s cluster. In contrast, residual vector quantization generates a global partition and then embeds it into each previous level’s cluster. It is similar to the hamming embedding (HE) method, while HE involves two levels and uses the orthogonal partition in each cluster. The simplified structure makes it possible to have more quantization levels and each level have more centroids for fine division of space. The method that transforming tree-like structure to flat structure, which has been used in ferns classifier [25], significant reduces the complexity of index structure while maintaining a fine-grained division of space.

### 5.3. Complexity

Processing vectors in original high dimensional space causes negative implications for complexity. Operations such as finding the nearest centroid or generating residual vectors are performed in high dimensional space while product quantization process subvectors in the low dimensional subspace. The memory usage of codebook is negligible when compared to the memory occupied by a codeddatabase. The complexity of look-up table computation is also negligible when compared with the complexity of scanning the database’s codes. The drawback is the computational complexities of learning and quantization stage of residual vector quantization are linear times of the complexities of product quantization. Our feature work will focus on reducing the complexities of learning and quantization stage.

## 6. Conclusions

We have introduced residual vector quantization for approximate nearest neighbor search. Two efficient search approaches are proposed based on residual vector quantization. The non-exhaustive search method significantly improves the performance. We evaluate the performance on two structured datasets and one unstructured dataset, and compare our approaches with spectral hashing and product quantization. Our approaches obtain the best results in terms of the trade-off between accuracy, speed and memory usage. Results on structured datasets show our approaches slightly outperform product quantization. For unstructured data, our approaches significant outperform the product quantization.

## Figures and Tables

**Table 1.** Dataset information.

**Dataset**	**SIFT**	**GIST**	**VLAD**
Dimension of descriptor	128	960	128
Size of learning set	100,000	500,000	1,491
Size of database set	1,000,000	1,000,000	1,491
Size of query set	10,000	1,000	500

**Table 2.** Comparison of RVQ and IVFRVQ on SIFT dataset.

**Method**	**Parameters**	**Search time(msec)**	**Average number of scanned codes**	**Recall @100**
RVQ	*L =* 8, *K =* 256	34	1,000,000	0.96
IVFRVQ	*L*_1_*=* 1, *L*_2_*=* 8, *K =* 256,*W =* 1	0.65	4,261	0.56
	*L*_1_*=* 1, *L*_2_*=* 8, *K =* 256,*W =* 8	**2.6**	**33,602**	**0.93**
	*L*_1_*=* 2, *L*_2_*=* 8, *K =* 256,*W =* 64	3.2	1,682	0.80
	*L*_1_*=* 2, *L*_2_*=* 8, *K =* 256,*W =* 512	15.1	9,692	0.96

**Table 3.** Comparison of RVQ and IVFRVQ on GIST dataset.

**Method**	**Parameters**	**Search time(msec)**	**Average number of scanned codes**	**Recall @100**
RVQ	*L =* 8, *K =* 256	36.1	1,000,000	0.67
IVFRVQ	*L*_1_*=* 1, *L*_2_*=* 8, *K =* 256,*W =* 1	2.9	5,205	0.36
	*L*_1_*=* 1, *L*_2_*=* 8, *K =* 256,*W =* 8	**4.6**	**42,699**	**0.67**
	*L*_1_*=* 2, *L*_2_*=* 8, *K =* 256,*W =* 64	5.7	2,423	0.55
	*L*_1_*=* 2, *L*_2_*=* 8, *K =* 256,*W =* 512	20.5	16,512	0.74

**Table 4.** Comparison with state of the art on VLAD dataset.

	
	**32 bits**	**64 bits**	**128 bits**
SH	0.255	0.349	0.397
PQ	0.337	0.409	0.457
RVQ	**0.407**	**0.510**	

**Table 5.** Search speed for 64-bit code and different methods (SIFT dataset).

**Method**	**Parameters**	**Search time(msec)**	**Average number of scanned codes**	**Recall @100**
RVQ	*L* = 8, *K* = 256	34	1,000,000	0.96
***IVFRVQ***	*L*_1_ = 1, *L*_2_ = 8, *K* = 256,*W* = 8	***2.6***	33,602	***0.93***
PQ	*L* = 8, *K* = 256	33.7	1,000,000	0.93
IVFPQ	*K*′ = 1024, *L* = 8, *K* = 256,*W* = 8	3	9,102	0.87
IVFPQ	*K′* = 1024, *L* = 8, *K* = 256,*W* = 16	7.3	17,621	0.93
SH	*nbit* = 64	35.3	1,000,000	0.53
